# The effect of a loading dose of meropenem on outcomes of patients with sepsis treated by continuous renal replacement: study protocol for a randomized controlled trial

**DOI:** 10.1186/s13063-022-06264-2

**Published:** 2022-04-12

**Authors:** Sui-Qing Ni, Wen-Bing Teng, Yong-Hong Fu, Wei Su, Zhi Yang, Jie Cai, Jin-Nuo Xu, Xiao-Ying Deng, Xiang-Fang Liu, Sheng-Nan Fu, Jun Zeng, Chen Zhang

**Affiliations:** 1 Department of Pharmacy, Guangzhou First People’s Hospital, School of Medicine, South China University of Technology, Guangzhou, 510180 Guangdong China; 2 Department of Critical Care Medicine, Guangzhou First People’s Hospital, School of Medicine, South China University of Technology, Guangzhou, 510180 China

**Keywords:** Sepsis, Continuous renal replacement therapy (CRRT), Meropenem, Loading dose, Pharmacokinetic (PK)/pharmacodynamics (PD) target, Clinical cure rate, Bacterial clearance rate

## Abstract

**Background:**

Sepsis and continuous renal replacement therapy (CRRT) are both responsible for the alterations of the pharmacokinetics of antibiotics. For patients with sepsis receiving CRRT, the serum concentrations of meropenem in the early phase (< 48 h) was significantly lower than that in the late phase (> 48 h). This current trial aimed to investigate whether administration of a loading dose of meropenem results in a more likely achievement of the pharmacokinetic (PK)/pharmacodynamics (PD) target (100% fT > 4 × MIC) and better therapeutic results in the patients with sepsis receiving CRRT.

**Methods:**

This is a single-blinded, single-center, randomized, controlled, two-arm, and parallel-group trial. This trial will be carried out in Guangzhou First People’s Hospital, School of Medicine, South China University of Technology Guangdong, China. Adult patients (age ≥ 18 years) with critical sepsis or sepsis-related shock receiving CRRT will be included in the study. The subjects will be assigned to the control group and the intervention group (LD group) randomly at a 1:1 ratio, the estimated sample size should be 120 subjects in each group. In the LD group, the patient will receive a loading dose of 1.5-g meropenem resolved in 30-ml saline which is given via central line for 30 min. Afterward, 0.75-g meropenem will be given immediately for 30 min every 8 h. In the control group, the patient will receive 0.75-g meropenem for 30 min every 8 h. The primary objective is the probabilities of PK/PD target (100% fT > 4 × MIC) achieved in the septic patients who receive CRRT in the first 48 h. Secondary objectives include clinical cure rate, bacterial clearance rate, sepsis-related mortality and all-cause mortality, the total dose of meropenem, duration of meropenem treatment, duration of CRRT, Sequential Organ Failure Assessment (SOFA), C-reactive protein levels, procalcitonin levels, white blood cell count, and safety.

**Discussion:**

This trial will assess for the first time whether administration of a loading dose of meropenem results in a more likely achievement of the PK/PD target and better therapeutic results in the patients with sepsis receiving CRRT. Since CRRT is an important therapeutic strategy for sepsis patients with hemodynamic instability, the results from this trial may help to provide evidence-based therapy for septic patients receiving CRRT.

**Trial registration:**

Chinese Clinical Trials Registry, ChiCTR2000032865. Registered on 13 May 2020, http://www.chictr.org.cn/showproj.aspx?proj=53616.

**Supplementary Information:**

The online version contains supplementary material available at 10.1186/s13063-022-06264-2.

## Background

Critical sepsis and sepsis-related shock are common in intensive care units (ICUs) which result in high mortality and morbidity [1, 2]. Acute kidney injury (AKI), as a common complication of serious sepsis or sepsis-related shock, is a tricky issue in those who are critically ill in the ICU [3]. In ICU, the occurrence of AKI is 16–65% [4, 5]. Renal replacement therapy (RRT) is a common treatment for these patients, and about 70% of them require this therapy [4]. There are various types of renal replacement therapies and continuous renal replacement therapy (CRRT) is the most commonly adopted in ICU because of its benefits in patients with unstable hemodynamics in comparison with the intermittent methods [6]. In patients with sepsis, CRRT is more often preferred to conventional RRT for it is better tolerated hemodynamically [7]. Applying antimicrobial treatment in an early stage is the key step in treating critical sepsis and sepsis-related shock [5, 7]. The mortality of patients with sepsis-related shock will significantly increase if the administration of the antibiotics is delayed [8, 9]. According to the Surviving Sepsis Campaign guidelines 2016, it was recommended that effective antibiotics should be given to the patients in 1 h after critical sepsis or sepsis-related shock was diagnosed [10].

As a broad-spectrum carbapenem, meropenem can prevent anaerobic, Gram-negative, and Gram-positive microorganisms, which is frequently applied as an empirical treatment for critical sepsis or sepsis-related shock [11]. It belongs to the β-lactam family. The function of antibiotics in the ß-lactam family relies on the time when the free [f] plasma drug concentration is higher than the minimal inhibitory concentration (fT > MIC) against the susceptible microorganisms [12]. It was considered that fT > MIC should be 40 to 50% of the dosage interval at least. When fT > MIC is 60 to 70%, the killing effect will reach the maximum [13]. Ideally, the concentration of the free drug is 4–6 times above the MIC and the duration should be 40% of the dosing interval time at least, which depends on the kind of β-lactam used by the patients [14–16]. No other benefits or an elevation of the antibacterial activity is shown even if the concentrations of β-lactam drugs in serum are higher than these values [17]. However, considering that ICU patients are severely vulnerable to suboptimal doses and represent a source of selection of (multi) resistance to antibiotics, experts advocate a target concentration of 100% fT ≥ 4 × MIC as the pharmacokinetic(PK)/pharmacodynamic(PD) target [18]. Meropenem is a small molecule featured as hydrophilic nature and low distribution volume (V; 0.3 L/kg). Its protein binding level is extremely low (< 2%) [19]. These characteristics make meropenem susceptible to be removed by the kidney and CRRT [20].

It is paramount to obtain the concentrations of the target antibiotics with empirical strategies of dosing in treating the septic patients receiving CRRT. However, this is complex in septic patients receiving CRRT because of the critical condition and extracorporeal circuit. The pharmacokinetics of the antibiotics may change, and thus, attaining these pharmacodynamic targets may be difficult [21]. Low concentrations of the antibiotics might cause the failure of the treatment and resistance against antimicrobial drugs [22, 23]. High concentrations of beta-lactam drugs might contribute to toxicity, especially neurotoxicity [24]. Most studies have concluded that the dose should be adjusted in those who receive CRRT. Seyler et al. reported that for patients with sepsis receiving CRRT, the serum concentration of meropenem in the early phase (< 48 h) was significantly lower than that in the late phase (> 48 h) (Student’s *t* test, *P* = 0.018) [25]. Drusano et al. demonstrated that a higher dosing (e.g., loading dose) at the first time can prevent pathogen inoculum in the early stage during treatment, which may facilitate the immune system of the host to remove the pathogens [26]. Therapeutic drug monitoring (TDM) at trough levels can be useful in adjusting dosing strategies in the maintenance period during treatment [27]. Hagel et al. demonstrated that continuous infusion of tazobactam/piperacillin with TDM guidance in septic patients leads to a decreased incidence of organ dysfunction and better therapeutic results in comparison with the infusion without TDM guidance [28].

To conclude, increasing evidence has presented that changed pharmacokinetics of the septic patients receiving CRRT resulted in an inappropriate exposure to the antibiotics, which led to negative effects of treatment. By applying an initial loading dose, TDM-guided therapy, the PK/PD target (100% fT > 4 × MIC) may be more likely achieved in septic patients receiving CRRT, and clinical outcomes may be improved.

## Methods

### Trial objectives

The most important aim is to assess if PK/PD target (100% fT > 4 × MIC) can be more likely achieved and better clinical outcomes can be obtained in patients with sepsis receiving CRRT when a loading dose of meropenem is applied.

### Study design

**Fig. 1:**
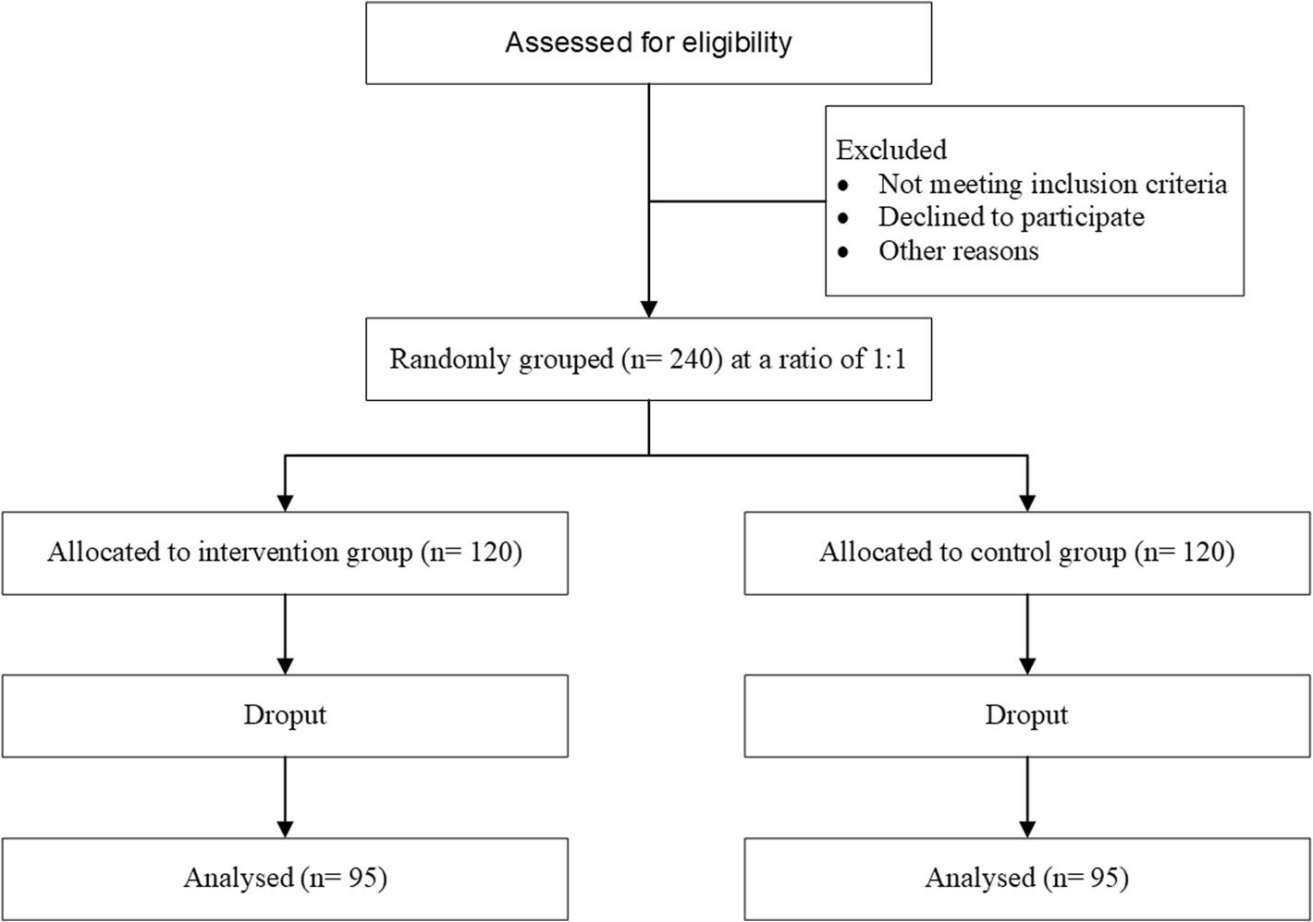
Flow diagram of subject enrollment, intervention strategies, and evaluation

This is a single-blinded (trial participants), single-center, randomized, controlled, two-arm, and parallel-group trial for assessing the PK/PD and clinic benefits of a loading dose of meropenem in patients with sepsis receiving CRRT. The trial will be carried out in Guangzhou First People’s Hospital, School of Medicine, South China University of Technology, Guangdong, China. The plan of subject enrolment, interventions strategies, and evaluation is presented in Fig. 1 and Table 1. Ethical approval has been obtained from the Guangzhou First People’s Hospital Ethics Committee. A Standard Protocol Item: Recommendations for Interventional Trials (SPIRIT) checklist is available in online supplementary file 1. Auditing is undertaken to check the compliance of the present studies. Any protocol amendment will be submitted to the ethical board for approval. Our research was registered at the Chinese Clinical Trials Registry (ChiCTR2000032865).

**Table 1:** Schedule of subject enrollment, intervention strategies, and evaluation

	STUDY PERIOD																
		Day after randomization															
Time point	-t1	0	1	2	3	4	5	6	7	8	9	10	11	12	13	14	EOT/E
**Enrolment:** **(May 2020-May 2021)**																	
Eligibility screen	×																
Informed consent	×																
Allocation		×															
APACHE II Score		×															
**Intervention:** **(May 2020-May 2021)**																	
Intervention group			×	×	×	×	×	×	×	×	×	×	×	×	×	×	
Control group			×	×	×	×	×	×	×	×	×	×	×	×	×	×	
**Assessments:** **(May 2020 -Dec 2021)**																	
PK/PD target			×	×													
Clinical response/ Microbiological outcomes					×		×		×			×				×	×
SOFA-score		×	×	×	×	×	×	×	×	×	×	×	×	×	×	×	×
CRP, WBC, PCT		×			×		×		×			×				×	×

*EOT* End of therapy, *E* Discharge from ICU, *SOFA* Sequential Organ Failure Assessment, *CRP* C-reactive protein, *WBC* White blood cell, *PCT* Procalcitonin

### The criteria of eligibility

#### Inclusion criteria

The criteria of inclusion are listed as follows:

- Age ≥ 18
- Critical sepsis or sepsis-related shock and receiving CRRT
- Meropenem will be used to treat infection
- Written informed consent obtained from the patients or their representatives

#### Exclusion criteria

The criteria of exclusion are listed as follows:

- Age < 18
- Pregnancy/lactation
- Hypersensitivity or allergy to meropenem
- Only using supportive or palliative therapies when eligibility was being assessed
- Liver dysfunction (Child-Pugh C)
- Participating who are involved in another clinical trial
- The measurement of meropenem is not available within 24 h following randomization

#### Dropout criteria

The dropout criteria are as follows:

- Withdrawn of the consent
- Severe adverse events
- Deceased

### Recruitment

The attendings will carry out the screening and subject inclusion.

### Randomization

Subjects are assigned randomly to the control group or the intervention group (LD group) at a 1:1 ratio. SPSS 17.0 will be used to create the randomization sequence by a physician who does not participate in the treatment or evaluation. The physicians and the nurses who give the treatment will not be blinded to the protocol allocation, but the subjects and the researchers who evaluate the outcomes will be blinded to the protocol allocation.

### Interventions

Continuous venovenous hemodiafiltration (CVVHDF) is the most common CRRT mode in our hospital. According to previous research, there are two different therapeutic schedules of meropenem, which are equally applicable to patients receiving CVVHDF: either 750 mg tid or 1500 mg bid [29]. Therefore, the former is selected as the administration scheme of the control group and the maintenance administration scheme of the LD group in our study. In the LD group, the patient will receive a loading dose of 1.5-g meropenem resolved in 30-ml saline which is given via central line for 30 min. Afterward, 0.75-g meropenem will be given immediately for 30 min every 8 h. In the control group, the patient will receive 0.75-g meropenem for 30 min every 8 h. Routine TDM is applied in the two groups once a day (at least from Monday to Friday) with notification of the results and adjustment of the dose on the same day if necessary. The dose can be adjusted by using the ratio equation because the kinetics of the test substance is linear by the physicians. Nonetheless, the adjustment of the dose should always consider other parameters such as the MIC of pathogenic bacteria or the recovery of kidney function.

### Renal replacement therapy

The septic patients who receive CVVHDF should be included in the study. Prisma CRRT systems will be applied. A sodium methallyl sulfonate copolymer filter, 1.5-m^2^ surface-treated acrylonitrile, sodium methallyl sulfonate copolymer filter, and 0.9-m^2^ acrylonitrile will be applied. All the settings of CRRT will be determined by the physicians who give the treatment.

### Antimicrobial therapy

Additional antimicrobials are used as combination therapy in the study. The attendings are responsible for carrying out the therapy. During the trial, de-escalation or escalation of the antimicrobial treatment is permitted at any time.

### Sample analysis

Blood samples are stored in the vials containing ethylenediaminetetraacetic acid (EDTA). All samples will be kept in a 4 °C refrigerator for 24 h. The selected blood samples are centrifuged and filtrated to determine free fractions. Afterward, the plasma samples are kept in the − 80 °C refrigerator. The determination of meropenem concentration is carried out in the study center by using validated high-performance liquid chromatography (HPLC).

### Primary objective

The primary objective is the probabilities of PK/PD target (100% fT > 4 × MIC) achieved in the septic patients who receive CRRT in the first 48 h. MIC data is established according to the data of susceptibility for isolated pathogens. When MIC data are obtained and for the subjects without isolated pathogen, the MIC breakpoint of *Acinetobacter baumannii* (4 mg/L) will be taken into consideration in preparing the “worst-case scenario”.

### Secondary objectives

The secondary objectives are listed as follows:

1. Clinical cure rate: Clinical response is assessed when the therapy stops and is recorded as failure or success. Success is considered as partial or complete recovery (improvement or cure) of temperature, leukocytosis, and clinical symptoms and signs associated with the infection. Failure is considered if (1) signs and symptoms of infection persist or deteriorate, (2) additional antibiotic therapy is required, or (3) death from infection. Two independent researchers will determine the condition if there is any doubt in judging the situation. A third physician will arbitrate the result if the two researchers cannot reach an agreement.
2. Bacterial clearance rate: The microbiological outcomes are evaluated by a microbiology specialist. Presumed eradication or eradication is considered as success. Resistance, persistence, and presumed persistence are considered as failure. For those who have various organisms on the same site of infection, the organisms which are susceptible to meropenem will be used. When a new pathogen is detected at the infection site during meropenem treatment, the result is considered as colonization if it is unnecessary to use new antibiotics. Otherwise, the result is considered as superinfection if new antibiotics should be used. Colonization is categorized as success; however, superinfection resulted from the pathogens belonging to the therapeutic spectrum of meropenem is deemed as failure. Superinfections induced by *Candida* spp., *Enterococcus faecium*, other fungal species, and methicillin-resistant staphylococci (MRSA) are not categorized as failure. A clinician and a microbiologist will decide whether the result is colonization or superinfection. Evaluation for microbiological outcomes and clinical response will be performed in all included patients at several time points including the 3rd, 5th, 7th, 10th, and 14th day following randomization, the end of the treatment (EOT) with meropenem (for patients who stay in ICU more than 14 days for extended medical reasons), and the day when patients are discharged from ICU (who are discharged before the 14th day).
3. Sepsis-related mortality and all-cause mortality.
4. Total dose of meropenem and duration of meropenem treatment.
5. Number of days with CRRT.
6. Sequential Organ Failure Assessment (SOFA) scores.
7. Inflammatory biomarkers: C reactive protein (CRP), procalcitonin (PCT), and white blood cell (WBC) count are determined at the beginning and the end of meropenem treatment.
8. Safety: The safety is assessed by treatment-emergent adverse events according to clinical symptoms (seizures, vomiting, rash, and diarrhea), parameters in laboratory tests, and their variations during meropenem treatment (thrombocytes, bilirubin, alkaline phosphatase, and transaminases).

### Adverse events (AEs) and serious adverse events (SAEs)

There are various aberrations of the laboratory tests, and signs and symptoms are abnormal in septic patients because of the severity of the illness and the standard medicine treatment. AEs are not constituted by these aspects unless they are regarded as a concern in the investigator’s clinical judgment or associated with the therapy. AEs are defined as the events associated with study therapy (definitely possibly, or probably). Serious AEs are any accidental medical event that meets at least more than one criterion as follows: (1) death, (2) life-threatening events, (3) requiring hospitalization or prolonged hospitalization, (4) significant or persistent incapacity/disability, (5) birth defect/congenital anomaly.

The physician is in charge of judging the causal relationship of SAE as definitely not, possibly not, possibly, probably, or definitely related to the study treatment.

All the SAEs and AEs associated with the treatment will be recorded on eCRF. The Institutional Ethics Committee should be informed of SAEs within 24 h after the researcher notices the events.

### Data collection

Detailed data that are used to evaluate the baseline characteristics of the patients (inclusion and exclusion criteria, laboratory results, vital signs, disease severity, scores of organs dysfunction, concurrent clinical conditions, demographics, comorbidities, and sepsis diagnosis) and outcomes (e.g., ICU mortality, hospital mortality) will be collected by trained staff, and data will be entered into a web-based clinical trial database system (iMedidata). The database will contain validation scopes to minimize the chance of data entry errors. Missing data or suspicious errors will be resolved before database lock and analysis. Data are confidential, and only the principal investigator, statisticians, data manager, and other researchers will have access to the final trial data set.

### Safety monitoring plan

The Data Safety Monitoring Board (DSMB), which is independent of the present trial, is composed of three members, including two specialists in infectious diseases and one independent statistician with statistical support. It will monitor the performance of the overall study, ensure participants receive good clinical care and safety concerns, and review quality of the data through regular meetings. Moreover, the DSMB will make recommendations and decisions on continuation, modification, or termination of the study based on the interim analysis of safety and efficacy. The interim analysis of this trial is free from the conflict of interest, while considering the safety, cost, resources, and the meaning of the study. The appropriate alpha value of primary outcomes in each interim analysis will be calculated by the O’Brien-Fleming Spending Function. If a predictive probability of reaching a significant difference (one-sided alpha set as 0.001) between LD group and control group on mortality with the current number of the subjects exceeds 90%, this study will be stopped [30].

### Statistical analysis

#### Sample size determination

In DALI research [31], 60% subjects have not reached PD target. An assumption is made that it is possible to reduce this percentage to 40%. The size of the sample is calculated when *α* is 0.05 (two-sided test) and *β* is 0.2. By applying PASS 15 software (NCSS Statistical Software, Kaysville, UT, USA) for the calculation, the results show that 95 subjects should be included in each group. If the attrition rate is less than 20%, the number of suitable subjects should be 119 at least in each group. Thus, there should be 120 subjects in each group (*n* = 240).

#### Data analysis

The primary endpoint will be analyzed following the intention-to-treat (ITT) principle. *P* value less than 0.05 is regarded as statistically significant. The missing values will be carefully reviewed and be handled with multiple imputation [32]. All tests are two-sided without adjustment in multiple comparisons. Continuous values are presented as interquartile ranges, average values, and medians or standard deviations. Categorical values are presented as proportions. For dichotomous efficacy endpoints, risk ratios and risk differences with 95% confident intervals are calculated. Multiplicity adjustments will be applied for chi-square endpoints and subgroup analysis.

Secondary endpoints will be analyzed. Rates of the two groups are compared by using Fisher’s exact test or chi-square test. Relative and absolute frequencies are used to report adverse events in each group. Student’s *t* test or nonparametric test is used to compare means between two groups.

### Rules for termination

This study can be discontinued ahead of schedule when any situation listed below occurs: (1) the patients or their representatives retract their informed consent, (2) inadequate protocol adherence is repeatedly observed in participants or investigators, (3) data are lack of quality or insufficient recruitment, and (4) the study medication has critical side effects; it is detrimental for the patients to participate in the trial.

### Patient and public involvement

This study was designed without patient involvement. Patients were not invited to comment on the study design and were not consulted to develop patient-relevant outcomes. Patients were not invited to contribute to the writing or editing of this document for readability or accuracy. Ancillary and post-trial care was provided by the participated physicians.

### Ethics and dissemination

All trial participants have obtained Good Clinical Practice certification. This research protocol was approved by the ethics committee of Guangzhou First People’s Hospital, School of Medicine, South China University of Technology (ref. K-2017-067-03), and registered at the China clinical trial registration center. All subjects or their legal representatives should provide written informed consent. To ensure antibiotic therapy in the early stage, the ethics committee approved a provision that acquiring the consent can be delayed as critically ill patients are often incapacitated. For participants enrolled under this provision, the subjects’ representatives should provide the informed consent as soon as possible following enrollment. Otherwise, the patient should be excluded. Results will be disseminated directly to study participants at the end of the trial and to other stakeholders via publication in a peer-reviewed journal.

## Discussion

Promptly reaching the therapeutic concentrations of antibiotics is of great importance for those who have a severe infection in ICU. Inappropriate dosing can contribute to unfavorable infections [18, 33]. One method to prevent underdosing and increase antimicrobial concentrations is administration of an initial loading dose. Our research will explore whether administration of a loading dose of meropenem results in a more likely achievement of the PK/PD target and better therapeutic results in the patients with sepsis receiving CRRT. Better dosing method may contribute to better attainment of antibiotics plasma level target, which could lead to improved clinical outcomes [34].

The primary objective is set as the probabilities of the PK/PD target achieved in the first 48 h according to the relationship between survival and antibiotic administration in the early stage [8]. This target is challenging because there are few preclinical or clinical studies proposing remarkably changing targets [34]. The purpose of this study is to primarily avoid underdosing which can lead to a high clinical risk in septic patients [34].

The research will explore some other questions, such as the effect of receiving a loading dose of meropenem on clinical cure rate and bacterial clearance rate. In addition to underdosing, the risk for meropenem associated toxicity will also be evaluated.

## Trial status

Recruitment for this study since May 2020 was expected to be completed in May 2021.

Currently, we are still recruiting participants. The protocol is version 2.1, 5 July 2020.

## Supplementary Information

**Additional file 1.** SPIRIT 2013 Checklist: Recommended items to address in a clinical trial protocol and related documents*.

## Data Availability

The full protocol will be available from the corresponding author after identification. Datasets generated or analyzed during the study will not be made public until they are published in a peer-reviewed international journal.

## Abbreviations

ICUs: Intensive care units
AKI: Acute kidney injury
RRT: Renal replacement therapy
CRRT: Continuous renal replacement therapy
LD: Loading dose
TZP: Tazobactam/piperacillin
Vd: Volume of distribution
TDM: Therapeutic drug monitoring
SPIRIT: Standard Protocol Item: Recommendations for Interventional Trials
EDTA: Ethylenediaminetetraacetic acid
CVVHDF: Continuous venovenous hemodiafiltration
HPLC: High-performance liquid chromatography
MRSA: Methicillin-resistant staphylococci
EOT: End of the treatment
CRP: C-reactive protein
WBC: White blood cell
PCT: Procalcitonin
AEs: Adverse events
SAEs: Serious adverse events
DSMB: Data Safety Monitoring Board
ITT: Intention-to-treat
